# New Appliances and Things Medical

**Published:** 1906-05-19

**Authors:** 


### New Appliances and Things Medical.

### VASELINE.

(Chesebrough Manufacturing Company, New York, and 42 Holborn Viaduct, London, E.C.)

The term "vaseline" has become so familiar that it is often erroneously identified with and applied to preparations which bear external resemblance to vaseline, but differ materially in substance. It is therefore well to know that the name "Vaseline" is the registered trade-mark of the Chesebrough Company, and applies only to their preparations. Vaseline is a semi-solid mixture of various hydrocarbons obtained from the undistilled residuum of light-gravity petroleum. As an emollient it possesses marked advantages over animal and vegetable fats, the chief perhaps being that it will keep indefinitely and does not become rancid, and it is excellent as a base for all kinds of ointments. Samples of the following have been brought to our notice: Ordinary vaseline and white vaseline, for medicinal and toilet purposes; vaseline cold cream and vaseline camphor ice, both of which are most agreeable and soothing toilet preparations; and capsicum-vaseline, which is a counter-irritant that does not blister the skin, is gentler in its action than a mustard-plaster, and therefore is a useful and convenient substitute. All these preparations of the Chesebrough Company are of excellent quality.

